# Effects of Multi-Frequency Ultrasound-Enhanced Subcritical Water on the Extraction of Phenolic Compounds from Rapeseed Meal

**DOI:** 10.3390/foods15203687

**Published:** 2026-10-16

**Authors:** Xiaoai Wang, Sheeza Rasheed, Jingyu Wei, Chenchen Yao, Yuqing Duan, Kai Hu, Dongyan Chen, Wenlin Li, Haihui Zhang

**Affiliations:** 1 School of Food Science and Engineering, Jiangsu University, Zhenjiang 212013, China; wangxaaa0129@163.com (X.W.); sheezarasheed078@gmail.com (S.R.); 19503888764@163.com (J.W.); 19552156117@163.com (C.Y.); kai.hu@ujs.edu.cn (K.H.); dongyan.chen@ujs.edu.cn (D.C.); 2 Institute of Food Physical Processing, Jiangsu University, Zhenjiang 212013, China; 3 Oil Crops Research Institute, Chinese Academy of Agricultural Sciences, Wuhan 430062, China; wenlinli2005@163.com

**Keywords:** rapeseed meal, polyphenols, ultrasound-assisted extraction, subcritical water extraction, in vitro antioxidant capacity

## Abstract

Conventional extraction methods for rapeseed meal polyphenols are limited by low yields and co-extraction of anti-nutritional factors. This study systematically evaluated the effects of subcritical water extraction (SWE), single-, dual-, and triple-frequency ultrasound-assisted extraction (SUE, DUE, TUE), and their combinations (SUSWE, DUSWE, TUSWE) on dry-extract yield, the composition and in vitro antioxidant capacity of the purified extracts, and the microstructure of the solid residues. Among all methods, DUSWE demonstrated superior performance. DUSWE achieved the highest dry-extract yield (4.60%), a 33.54% increase relative to conventional hot water extraction (HWE). Compared with the purified HWE extract, the purified DUSWE extract showed significantly higher total phenolic, total flavonoid, and anthocyanin contents but lower measured phytic acid and glucosinolate contents (*p* < 0.05). Scanning electron microscopy (SEM) showed DUSWE induced a porous microstructure favoring polyphenol release. Furthermore, it exhibited markedly higher in vitro antioxidant capacity. In summary, dual-frequency ultrasound (20/40 kHz) combined with subcritical water extraction significantly improves the yield and in vitro antioxidant capacity of rapeseed polyphenols, offering an effective strategy for high-value utilization of rapeseed meal.

## 1. Introduction

Rapeseed (*Brassica napus*) is one of the three major oil crops globally. According to the content of erucic acid and glucosinolate, it is classified into traditional rapeseed and “double-low” rapeseed. The Chinese Agricultural Industry Standard (NY/T 1795–2009) [1] defines double-low rapeseed as rapeseed containing no more than 5.0% erucic acid in the fatty acids of the oil and less than 45.0 μmol/g of total glucosinolates in the meal. Rapeseed meal is the by-product of oil extraction from rapeseed, with an annual global production of approximately 40 million tons [2]. Rapeseed meal is rich in protein, fat, minerals, vitamins, and polysaccharides [3], but its utilization is limited by anti-nutritional factors such as glucosinolates, phytic acid and polyphenols. Polyphenols in rapeseed meal account for 2–4%, significantly higher than that in other oil crops. Rapeseed polyphenols (RP) mainly exist in bound forms, along with small amounts of free phenolic acids and tannins. They form complexes with proteins, reducing digestibility, and contribute to bitterness and astringency. Consequently, the efficient recovery of phenolic constituents from rapeseed meal is important for improving its utilization and added value [4]. However, RP exhibits antioxidant, antitumor, anti-inflammatory, and antibacterial activities [5], highlighting the value of its extraction for both feed quality improvement and high-value product development.

In general, extraction methods are critical for phenolic compound research. Traditional extraction methods (e.g., reflux extraction, hot water extraction, and Soxhlet extraction) are easy to operate, but they generally suffer from issues such as long processing times, high solvent consumption, and low extraction efficiency. Furthermore, the extensive use of organic solvents (hexane and methanol) poses environmental and health hazards [6]. Green alternatives mainly include pulsed electric field [7,8], supercritical fluid [9,10], high hydrostatic pressure [11], microwave [12,13], subcritical water [14], and ultrasound-assisted extraction [15,16,17]. However, pulsed electric field requires high energy; supercritical fluid extraction involves high costs and is mainly for non-polar compounds; microwave-assisted extraction relies on polar solvents. Although SWE requires pressure-resistant equipment and involves high initial investment, it uses water as extraction solvent, thereby reducing the consumption and residual risks of organic solvents. Moreover, the tunable polarity of subcritical water facilitates the extraction of phenolic compounds with different polarities [18]. Under SWE conditions, water’s dielectric constant decreases, making it suitable for extracting components such as polysaccharides [19,20] and phenolic compounds [21], while reduced viscosity enhances mass transfer [22]. To further improve polyphenol yield and preserve bioactivity, ultrasound-assisted extraction (UAE) combined with SWE has been widely explored. Ultrasound promotes extraction through cavitation, which involves the formation, expansion, and collapse of bubbles in a liquid medium [23]. When bubbles collapse, shockwaves and micro-jets generate high local temperature and pressure, disrupting cell walls and enhancing solvent penetration and mass transfer [24]. Ultrasonic frequency is a key factor affecting cavitation intensity [25]. Compared with single-frequency ultrasound, dual- or multi-frequency ultrasound generates more bubbles, significantly increasing cavitation effects [26,27,28], and overcomes energy non-uniformity [29]. Yang et al. [30] found that dual-frequency ultrasound can enhance both the yield and quality of protein polypeptides from housefly larvae. Xu et al. [31] reported that tri-frequency ultrasound increased polyphenol yield from Pingyin rose by 19.33%. Zhang et al. [32] demonstrated that dual-frequency ultrasound significantly improved the functional properties of pea protein. Ultrasound integrating with pressurized extraction processes is a promising research direction. Barrales et al. [33] applied ultrasound in the supercritical CO_2_ extraction of passion fruit seed oil, resulting in 29% increase in the overall extraction yield. Guo et al. [34] continuously applied ultrasound during the extraction of cinnamon bark oil, whereas Kashtiban and Esmaiili et al. [35] employed ultrasound pretreatment followed by SWE to recover phenolic compounds from grape skin. However, previous studies primarily focused on single-frequency ultrasound and plant matrices other than rapeseed meal. The effects of different ultrasound-frequency pretreatments followed by SWE on the recovery and composition of phenolic compounds from rapeseed meal remain unclear.

Therefore, this study employed a two-step sequential process consisting of single-, dual-, or tri-frequency ultrasound pretreatment followed by SWE. The effects of different frequency modes on dry-extract yield, phenolic compounds, in vitro antioxidant capacity, anti-nutritional factors, and rapeseed meal microstructure were systematically compared. This study aims to clarify how ultrasound-frequency pretreatment affects the SWE of phenolic compounds from rapeseed meal and provide a basis for selecting an effective frequency mode.

## 2. Materials and Methods

### 2.1. Materials

Rapeseed meal (Variety: Huayou Jiaoyi 50) is the by-product of rapeseed oil by mechanical processing, which was provided by the Oil Crops Research Institute of the Chinese Academy of Agricultural Sciences (Wuhan, China). The sample was immediately ground and sieved through a 60-mesh sieve before extraction. The sieved powder was sealed in zip-lock bags and stored at −20 °C until use.

Chemicals and reagents: Sodium carbonate, sodium hydroxide, sodium nitrite, aluminum nitrate, hydrochloric acid, ferric chloride, sodium acetate, potassium persulfate, ethanol, and ammonium acetate were purchased from China National Pharmaceutical Group Chemical Reagent Co., Ltd. (Shanghai, China). AB-8 macroporous adsorption resin, Folin–Ciocalteu reagent, gallic acid, rutin, tannic acid, cyanidin, sodium phytate, sinigrin, sodium carboxymethyl cellulose, palladium chloride, 2,2-diphenyl-1-picrylhydrazyl (DPPH), 2,2′-azino-bis(3-ethylbenzothiazoline-6-sulfonic acid) diammonium salt (ABTS), and ferrous sulfate heptahydrate were purchased from Macklin (Shanghai, China). All other chemicals and solvents used in the experiments were of analytical or HPLC grade.

### 2.2. Equipment

Subcritical water reactor (Zhenjiang, China) and multifrequency ultrasound equipment (Self-developed by Jiangsu University, Zhenjiang, China) were used in this study. The ultrasound system comprised an approximately 7.4 L stainless-steel chamber equipped with six frequency-sweep generators and an external refrigerated circulator for temperature control. The frequency modes of 40, 20/40, and 20/40/60 kHz were operated continuously, with the dual- and tri-frequency modes applied simultaneously. SWE was performed using a static 25 mL batch reactor comprising a 304 stainless-steel outer vessel and a PTFE liner. The reactor was operated without mechanical agitation or external pressurization.

### 2.3. Preparation of Rapeseed Polyphenol Extracts

#### 2.3.1. Multifrequency Ultrasound Extraction of Crude Polyphenols

Crude RP was extracted as the method of Zhang et al. [36] with slight modifications. The rapeseed meal was mixed with distilled water at a ratio of 1:20 (g:mL), then treated with ultrasound (168 W, frequencies of 20, 40, and 60 kHz, respectively) at 20 °C for 20 min. After filtration under vacuum, the polyphenol content in the filtrate was determined by the Folin–Ciocalteu method, and the yield was calculated. Single-factor experiments were conducted to optimize extraction conditions, investigating the effects of ultrasound temperature (10 °C, 20 °C, 30 °C, 40 °C, 50 °C), time (10 min, 15 min, 20 min, 25 min, 30 min), power (120 W, 144 W, 168 W, 192 W, 216 W), and frequency (20 kHz, 40 kHz, 60 kHz, 20/40 kHz, 40/60 kHz, 20/40/60 kHz) on the yield of RP. The ultrasound single-factor experiments were conducted sequentially in the order of temperature, time, power, and frequency, with the selected condition from each step retained in the subsequent experiment. The fixed conditions were 20 min, 168 W, 40 kHz, and 1:20 (g:mL) when temperature was varied; 20 °C, 168 W, 40 kHz, and 1:20 (g:mL) when time was varied; 20 °C, 20 min, 40 kHz, and 1:20 (g:mL) when power was varied; and 20 °C, 20 min, 168 W, and 1:20 (g:mL) when frequency mode was varied.

#### 2.3.2. Subcritical Water Extraction of Crude Polyphenols

Crude RP was extracted according to the method with slight modifications [22,37]. A mixture of 0.75 g rapeseed meal powder and 15 mL distilled water was extracted in a subcritical water reactor at 160 °C for 15 min. After the reaction, the mixture was filtered under vacuum, and the polyphenol content in the filtrate was determined by the Folin–Ciocalteu method to calculate the yield. The filtrate obtained was used for subsequent purification and component analysis, and the solid residue was used for Scanning electron microscopy. Single-factor experiments optimized the conditions, examining the effects of extraction temperature (100 °C, 120 °C, 140 °C, 160 °C, 180 °C), time (5 min, 10 min, 15 min, 20 min, 25 min), and solid-to-liquid ratio (1:10, 1:15, 1:20, 1:25, 1:30 g:mL) on the yield of RP. The SWE single-factor experiments were conducted sequentially in the order of extraction temperature, holding time, and solid-to-liquid ratio, with the selected condition from each step retained in the subsequent experiment. The holding time and solid-to-liquid ratio were fixed at 15 min and 1:20 (g:mL) when temperature was varied; the temperature and solid-to-liquid ratio were fixed at 160 °C and 1:20 (g:mL) when holding time was varied; and the temperature and holding time were fixed at 160 °C and 15 min when the solid-to-liquid ratio was varied.

#### 2.3.3. Traditional Hot Water Extraction

Rapeseed powder (12 g) was mixed with deionized water at a solid-to-liquid ratio of 1:20 (*w*/*v*) in a 250 mL beaker. Extraction was performed at 100 °C for 20 min. The extract was vacuum-filtered to collect the filtrate.

#### 2.3.4. Purification of Rapeseed Polyphenols

The crude extracts were purified using AB-8 macroporous adsorption resin based on the general procedure described by Liu et al. [38]. For each independent SWE or sequential extraction batch, extracts from 16 times (0.75 g of rapeseed meal and 15 mL of water per run) were pooled. For all treatments, the entire 240 mL crude extract from each independent batch was divided into two 120 mL portions for separate purification. Briefly, the column was packed with 50 g of wet AB-8 resin, corresponding to a bed volume of 40 mL (1 BV). For each purification, 120 mL of crude extract (3 BV) was loaded onto the column at a flow rate of 2–4 mL/min. After sample loading, the column was washed with 4 BV (160 mL) of deionized water at 2–4 mL/min [until the effluent became nearly colorless] to remove highly polar impurities. The phenolic-enriched fraction was subsequently eluted with 4–5 BV of 70% (*v*/*v*) ethanol at 2–4 mL/min, and the eluate was collected. The two eluates from the same independent batch were combined, concentrated under reduced pressure, and freeze-dried for yield determination and subsequent compositional and antioxidant analyses. The same purification procedure was applied to the crude extracts obtained from all extraction treatments (Table 1).

### 2.4. Determination of Dry Matter Yield of Rapeseed Polyphenol Extracts

The rapeseed polyphenol extract was freeze-dried according to the method [39,40], and the yield (Y) of the extract was calculated as follows:

(1) $$Y(\%)=(m_{2}-m_{1})\times 100/m_{0}$$

where Y is the dry-extract yield (%), *m*_1_ is the mass of the empty culture dish (g), *m*_2_ is the total mass of the culture dish and freeze-dried extract (g), and *m*_0_ is the initial mass of rapeseed meal used for each independent batch (12 g).

### 2.5. Analysis of Active Compounds

#### 2.5.1. Total Phenolic Content

Total phenolic content (TPC) was determined using the modified method of Lu et al. [41]. Briefly, 0.4 mL of the extract solution (10 mg/mL) was mixed with 2.4 mL water and 0.4 mL Folin–Ciocalteu reagent. After 6 min, 1.6 mL 10.6% Na_2_CO_3_ was added and reacted for 60 min in dark. Absorbance was recorded at 760 nm. Gallic acid was used as the reference standard, with a calibration range of 0–100 μg/mL. The calibration equation was y = 0.0018x + 0.0486 (R^2^ = 0.9986). Results were expressed as mg GAE/g DW.

#### 2.5.2. Total Flavonoid Content

Total flavonoid content (TFC) was determined based on the aluminum chloride colorimetric method described by Mamy et al. [42], with modifications. Absorbance was recorded at 510 nm. Rutin was used as the reference standard. The calibration curve covered a concentration range of 0–60 μg/mL, with the regression equation y = 0.0034x + 0.0871 (R^2^ = 0.9975). Results were expressed as mg RE/100g DW.

#### 2.5.3. Anthocyanin Content

Anthocyanin content was determined using the modified method [43]. Briefly, 200 mg of dry-extract was mixed with 12 mL of a hydrochloric acid–ethanol mixture, ultrasonically extracted at 30 °C for 30 min, and centrifuged at 4000 r/min for 10 min. Then, 200 µL of the supernatant was taken, and its absorbance was measured at 520 nm. Cyanidin was used as the reference standard. The calibration curve covered a concentration range of 0–0.04 mg/mL, with the regression equation y = 33.287x + 0.0424 (R^2^ = 0.9999). Anthocyanin content was expressed as mg/100 g.

### 2.6. Determination of Anti-Nutritional Factor Content

#### 2.6.1. Tannins

Tannin content was determined using a modified method [44,45]. Briefly, 0.2 mL of the extract solution (10 mg/mL) was mixed with 1.0 mL water and 0.4 mL Folin–Denis reagent. At last, 0.6 mL 75 g/L Na_2_CO_3_ was added and reacted for 40 min in dark. Tannic acid was used as the reference standard. The calibration curve covered a concentration range of 0–0.12 mg/mL, with the regression equation y = 4.826x + 0.0804 (R^2^ = 0.9991). Absorbance was measured at 765 nm, and total tannin content was expressed as mg/g.

#### 2.6.2. Phytic Acid

Phytic acid content was determined using the modified method [46,47]. Briefly, 350 mg of dry-extract was extracted with 7 mL of 2.4% HCl at 210 rpm for 2 h and centrifuged at 5000× *g* and 10 °C for 20 min. The supernatant was mixed with 0.7 g NaCl, held at −20 °C for 20 min, and centrifuged again before reaction with 15-fold diluted Wade reagent (0.3% FeCl_3_·6H_2_O and 3% sulfosalicylic acid) for 20 min. Sodium phytate was used as the reference standard. The calibration curve covered a concentration range of 0–0.10 mg/mL, with the regression equation y = −3.126x + 0.4151 (R^2^ = 0.9953). Absorbance was measured at 500 nm, and the total phytic acid content was expressed as mg/g.

#### 2.6.3. Glucosinolates

Glucosinolate content was determined using a modified method [48]. Endogenous enzymes in 200 mg of dry-extract powder were inactivated by heating in 2 mL of constant-temperature water at 75 °C for 5 min. Then, 5 mL of preheated 90% ethanol was added, and the mixture was heated at 60 °C for 6 min for extraction, with shaking every 2 min. The mixture was centrifuged at 5000 r/min for 5 min to collect the supernatant. Then, 0.2 mL of supernatant was mixed sequentially with 0.4 mL of sodium carboxymethyl cellulose and 0.2 mL of 8 mmol/L palladium chloride solution. After thorough mixing, the solution was incubated in a 27 °C water bath for color development for 2 h. Absorbance was measured at 540 nm against a palladium chloride blank solution. A standard curve was prepared using sinigrin. The calibration curve covered a concentration range of 0–1.20 µmol/mL, with the regression equation y = 0.2262x − 0.0502 (R^2^ = 0.9999). Total glucosinolate content was expressed in µmol/g.

### 2.7. Scanning Electron Microscopy (SEM)

Freeze-dried rapeseed meal residues from each treatment were mounted on SEM specimen stubs using conductive adhesive and sputter-coated with gold. Following Li et al. [24] with slight modifications, micrographs were acquired using a scanning electron microscope (S-3400N, Hitachi, Tokyo, Japan) at 1000× magnification and an accelerating voltage of 35 kV.

### 2.8. In Vitro Antioxidant Capacity

#### 2.8.1. DPPH Scavenging Activity

DPPH radical scavenging activity was determined following the method [49,50] with slight modifications. Briefly, 0.4 mL of the extract solution (25 mg/mL) was mixed with 2.6 mL of 0.1 mmol/L DPPH ethanol solution and kept in the dark for 30 min. Absorbance was measured at 517 nm using anhydrous ethanol as the blank control.

(2) $$DPPH\;radical\;scavenging\;capacity\;(\%)=(A_{0}-A_{1}+A_{2})/A_{0}\times 100$$

where A_1_ is the absorbance of the sample reaction mixture, A_2_ is the absorbance of the sample control, and A_0_ is the absorbance of the blank control.

#### 2.8.2. ABTS Scavenging Activity

ABTS radical scavenging activity was determined following the method [51,52] with slight modifications. The ABTS radical stock solution was stored at 4 °C for 12 h and then diluted with 95% ethanol until its absorbance at 734 nm reached 0.700 ± 0.020. Then, 0.2 mL of the extract solution (25 mg/mL) was mixed with 5.8 mL of the diluted ABTS solution and kept in the dark for 6 min. Absorbance was measured at 734 nm.

(3) $$\mathrm{ABTS}\;\mathrm{radical}\;\mathrm{scavenging}\;\mathrm{capacity}\;(\%)=(A_{1}-A_{2})/A_{1}\times 100$$

where A_1_ is the absorbance of the blank control and A_2_ is the absorbance of the sample control.

#### 2.8.3. FRAP

Ferric reducing antioxidant power (FRAP) was determined using the method [53,54] with slight modifications. Briefly, 0.2 g of dry-extract was mixed with 0.6 mL of water, followed by the addition of 6 mL of FRAP working solution (0.3 mol/L Sodium acetate buffer:10 mmol/L TPTZ:20 mmol/L FeCl_3_ = 10:1:1 (*v*/*v*/*v*). The mixture was incubated in a 37 °C water bath for 30 min, and absorbance was measured at 595 nm. The calibration curve covered a concentration range of 0–1 mmol/L, with the regression equation y = 3.8285x + 0.1593 (R^2^ = 0.9977).

### 2.9. Data Analysis

All independently replicated quantitative results were obtained from three separate extraction batches for each treatment or single-factor level (*n* = 3). Each independently prepared extraction batch was treated as one experimental unit. For analyses conducted using purified extracts, the three independently prepared extracts were purified, concentrated, and freeze-dried separately.

Data are expressed as mean ± standard deviation (SD). Each response variable was analyzed separately using a fixed-effect one-way analysis of variance (ANOVA), with extraction treatment or the investigated single-factor level included as the fixed factor. Normality of the model residuals and homogeneity of variance were evaluated using the Shapiro–Wilk and Levene tests, respectively. When the overall ANOVA was significant, the Waller–Duncan multiple-range test was used as the sole post hoc procedure to classify the means into homogeneous groups. Differences were considered significant at *p* < 0.05. Statistical analyses were performed using IBM SPSS Statistics 27.0 (IBM Corp., Armonk, NY, USA). Data were organized using Microsoft Excel 2016 (Microsoft Corp., Redmond, WA, USA), and figures were prepared using Origin 2024 (OriginLab Corporation, Northampton, MA, USA).

## 3. Results and Discussion

### 3.1. Single-Factor Experiments

#### 3.1.1. Parameter Selection for Ultrasound Extraction of Rapeseed Meal Polyphenols

**Figure 1:**
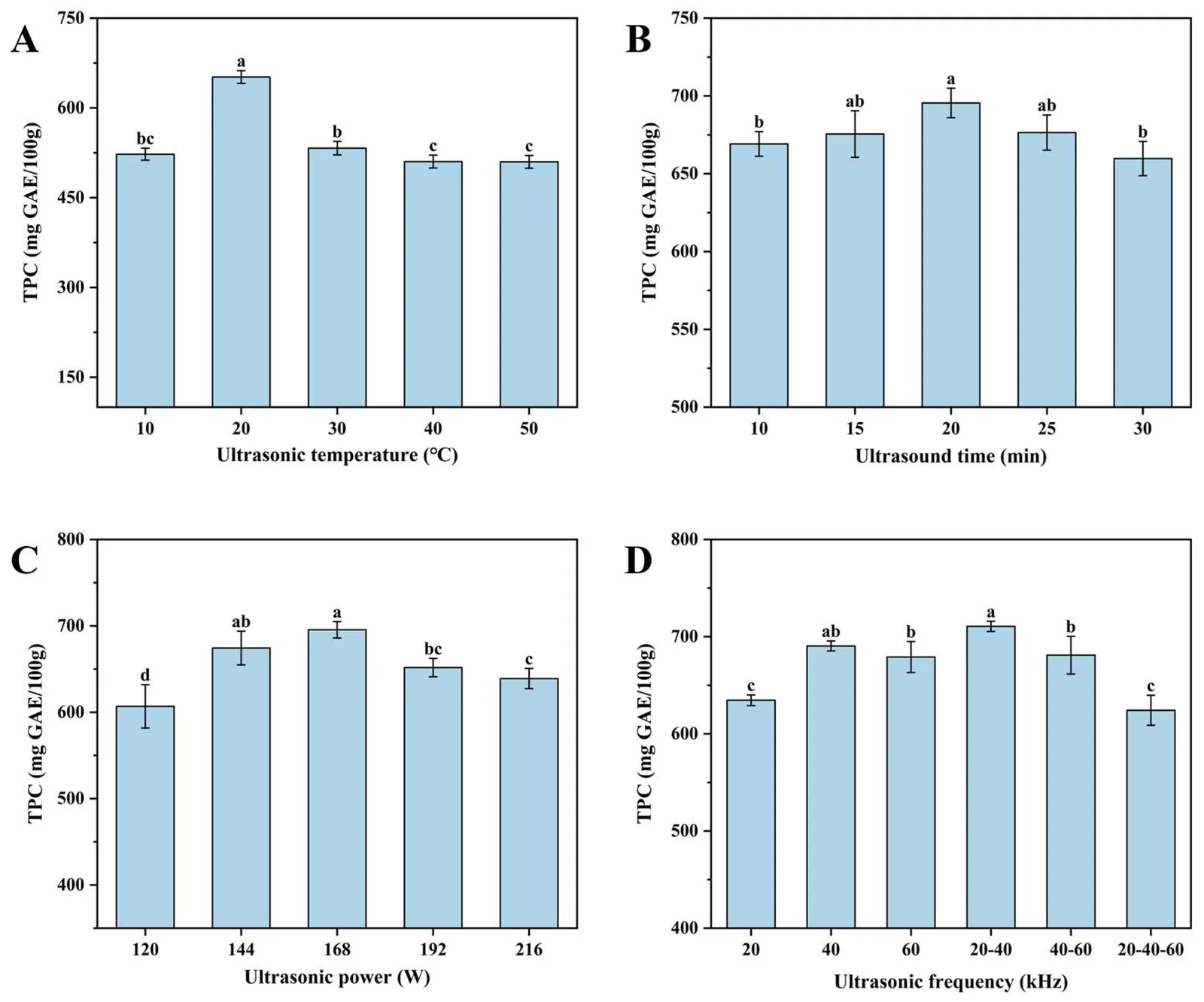
Effect of ultrasound temperature, time, power, and frequency on the content of crude polyphenols from rapeseed. (**A**) temperature; (**B**) time; (**C**) power; (**D**) frequency. Note: Data are presented as mean ± SD from three independent extraction batches (*n* = 3). Statistical differences were evaluated using one-way ANOVA followed by the Waller–Duncan multiple-range test. Different lowercase letters within the same panel indicate significant differences among treatments or factor levels (*p* < 0.05).

The effects of process parameters including ultrasonic temperature, time, power, and frequency on the TPC were investigated, as shown in Figure 1. The highest TPC (651.68 mg GAE/100 g) was achieved at 20 °C (Figure 1A). Increasing temperature may decrease surface tension and viscosity, enhancing the solubility of polyphenols from the matrix, thus promoting solvent penetration into the matrix and improving the extraction process [55]. When the temperature continued to increase beyond 20 °C, the TPC first decreased and then remained essentially constant. This is because higher temperatures may destabilize the molecular structure of polyphenols and accelerate oxidation [56]. The maximum TPC (695.49 mg GAE/100 g) was reached at 20 min (Figure 1B). Beyond 20 min, the TPC gradually decreased, which could be attributed to the thermal effect of ultrasound causing decomposition or transformation of some unstable rapeseed polyphenols. Within the studied ultrasonic power range, the TPC first increased and then decreased with increasing ultrasonic power, reaching a maximum (695.49 mg GAE/100 g) at 168 W, as shown in Figure 1C. Ultrasound can generate strong mechanical, cavitation, and thermal effects. The increase in power will enhance these effects, producing stronger pressure and micro-jets on the material and more rapidly and effectively disrupting the cell wall, promoting the release of more effective substances without damaging their structure and composition [57]. Due to the thermal effect of ultrasound, further increasing power generates stronger thermal effects, potentially leading to structural damage and reduced activity of thermosensitive components among the effective substances, which is consistent with the results of Xu et al. [31] on extracting polyphenols from Pingyin rose. Ultrasonic frequency is an important influencing factor [58,59] (Figure 1D). The TPC with dual-frequency ultrasound (710.59 mg GAE/100 g) was higher than that with single-frequency ultrasound (688.39 mg GAE/100 g) and tri-frequency ultrasound (624.25 mg GAE/100 g). The results indicate that dual-frequency ultrasound extraction can significantly improve the TPC [60], possibly because dual-frequency ultrasound extraction enhances the cavitation, thermal, and mechanical effects of ultrasound, leading to greater dissolution of polyphenols [61,62]. The superposition of more frequencies has both positive and negative effects on cavitation, thermal, and mechanical impacts [63]. Therefore, this may also explain why the TPC from tri-frequency ultrasound extraction was lower than that from dual-frequency extraction. Based on the above results, the temperature (20 °C), time (20 min), power (168 W), and frequencies (40 kHz, 20/40 kHz, 20/40/60 kHz) were selected for subsequent experiments.

#### 3.1.2. Parameter Selection for Subcritical Water Extraction of Rapeseed Meal Polyphenols

**Figure 2:**
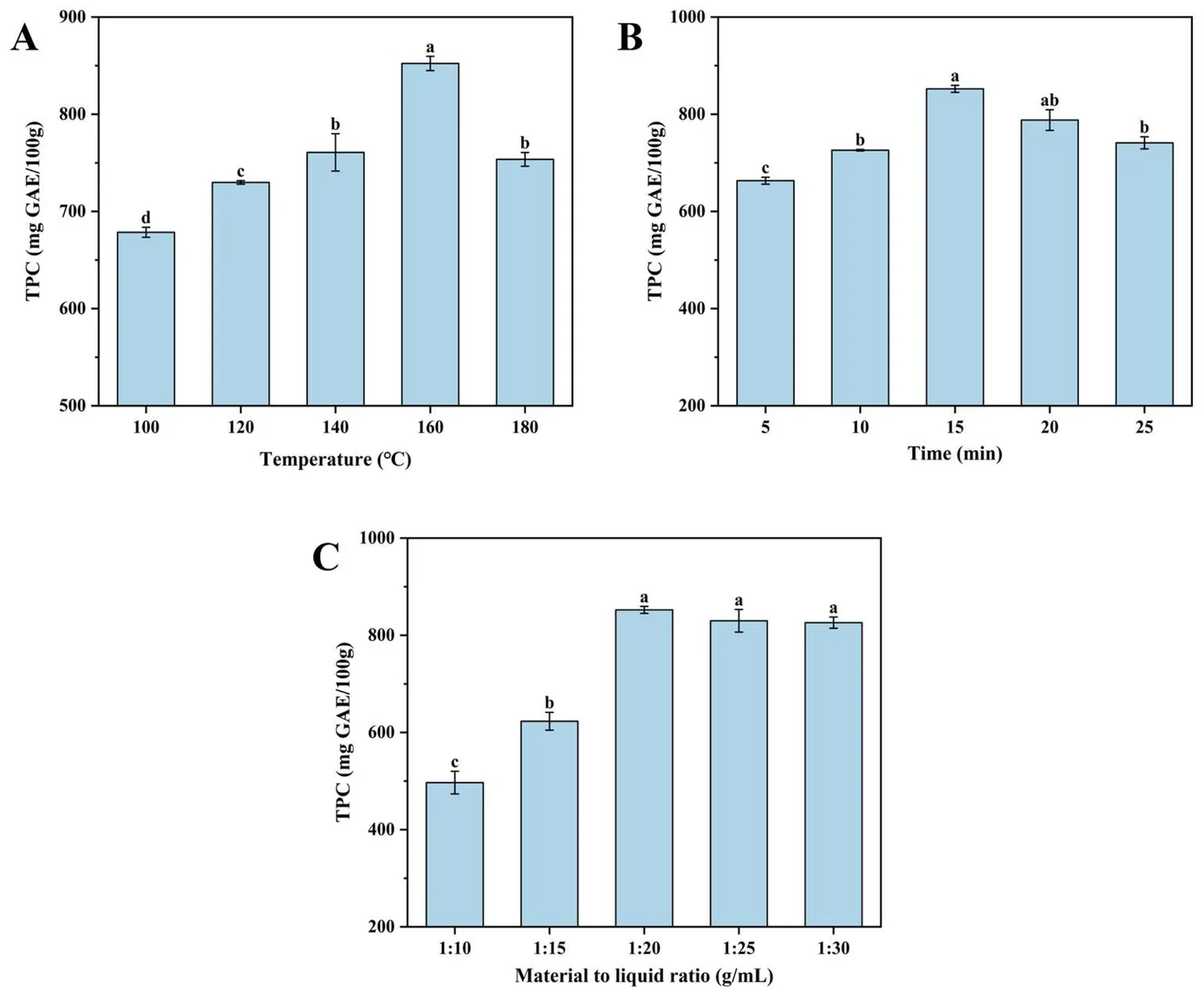
Effects of subcritical water temperature, time, and material-to-liquid ratio on the content of crude polyphenols from rapeseed. (**A**) temperature; (**B**) time; (**C**) material-to-liquid ratio. Note: Data are presented as mean ± SD from three independent extraction batches (*n* = 3). Statistical differences were evaluated using one-way ANOVA followed by the Waller–Duncan multiple-range test. Different lowercase letters within the same panel indicate significant differences among treatments or factor levels (*p* < 0.05).

Figure 2 shows the effects of temperature, time, and liquid-to-solid ratio on the TPC of rapeseed meal extracts obtained using SWE. The TPC increased with increasing time, temperature, and liquid-to-solid ratio, and decreased after reaching a certain threshold. During the subcritical extraction process, the interaction of these factors may enhance cell wall disintegration, solubilization, and diffusion of polyphenols into the extraction solvent, thereby accelerating the substantial transfer of extracts and effectively increasing TPC. This is consistent with the results of Chikari et al. [64] on extracting polysaccharides from Lentinus edodes.

Figure 2A shows that the highest TPC (852.25 mg GAE/100 g) was achieved at an extraction temperature of 160 °C. Increasing temperature enhances the solubility of phenolic compounds in water, thereby increasing TPC. However, when the temperature exceeded 160 °C, the TPC began to decrease, indicating that excessively high subcritical extraction temperatures cause degradation of less thermally stable polyphenols, resulting in a decreased content [65]. Figure 2B shows that within the range of 5–15 min, the TPC first increased with increasing time; within the range of 15–25 min, it decreased as time continued to increase. This is because the mass transfer is a solid–liquid equilibrium process, requiring a certain time to reach equilibrium [66]. As shown in Figure 2C, when the liquid-to-solid ratio reached 20:1 mL/g, the TPC was highest (852.25 mg GAE/100 g); as the liquid-to-solid ratio continued to increase, the TPC stabilized. When the liquid-to-solid ratio exceeded 20:1 mL/g, the rapeseed polyphenols were essentially fully extracted, and further increasing the ratio might lead to the co-extraction of other components from the raw material. Based on the above results, the extraction temperature (160 °C), extraction time (15 min), and liquid-to-solid ratio (20:1 mL/g) were selected for subsequent experiments.

### 3.2. Extraction Yield

**Figure 3:**
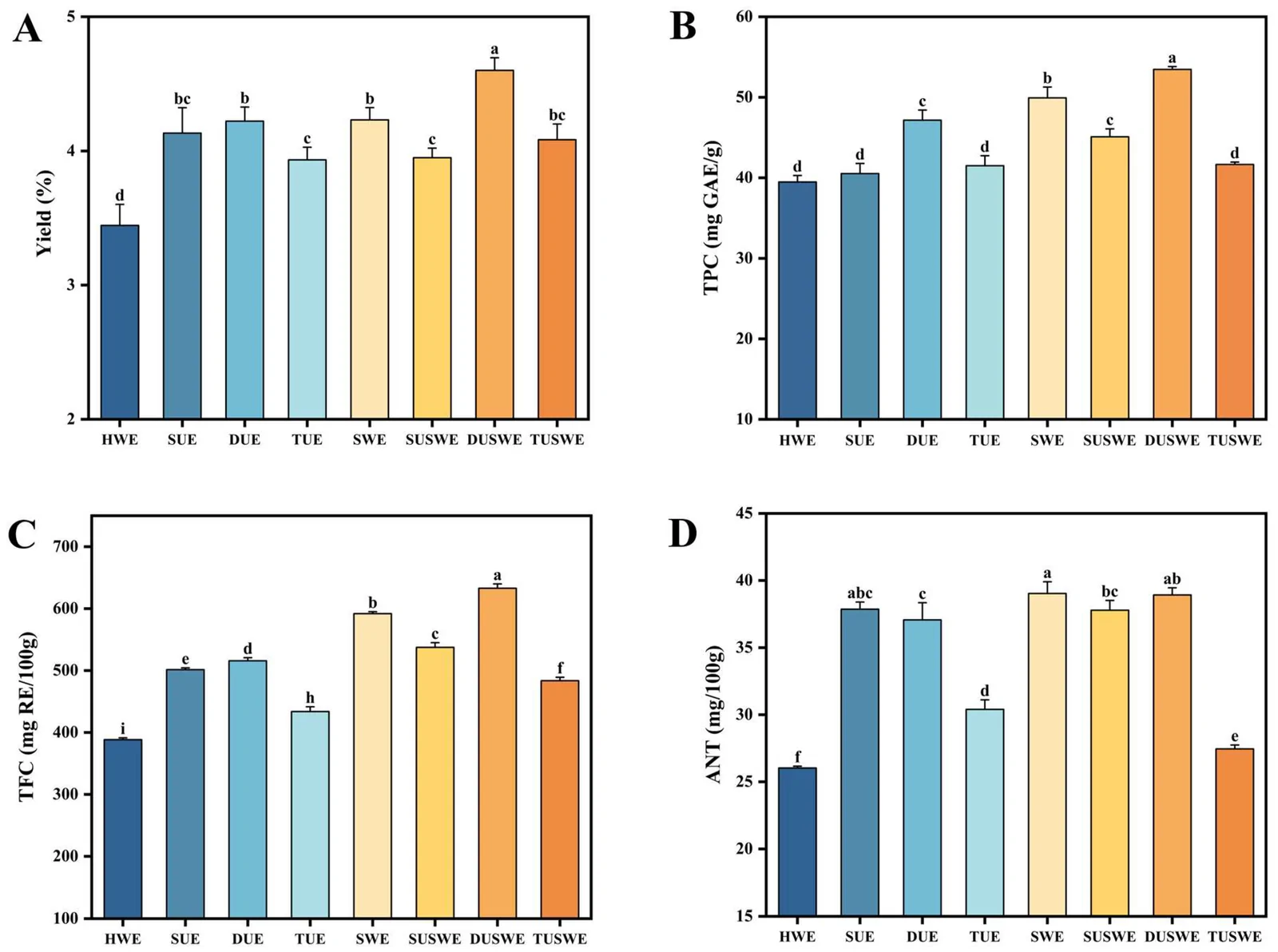
The effect of different methods on the content of active components. Note: (**A**) yield; (**B**) total phenolics; (**C**) total flavonoids; (**D**) anthocyanin. The values in panels B–D are expressed per unit mass of purified freeze-dried extract. Data are presented as mean ± SD from three independent extraction batches (*n* = 3). Statistical differences were evaluated using one-way ANOVA followed by the Waller–Duncan multiple-range test. Different lowercase letters within the same panel indicate significant differences among treatments or factor levels (*p* < 0.05).

Different extraction methods significantly affected the dry-extract yield (*p* < 0.05), as shown in Figure 3A. HWE yielded the lowest (3.44%), as its limited temperature cannot effectively disrupt the robust plant cell walls, dissolving only a small amount of easily accessible free polyphenols. Compared to HWE, SWE demonstrated a yield advantage. This is because, compared to HWE, SWE employs higher water temperatures and lower water polarity, which favor the dissolution of polyphenolic compounds from the matrix [67]. Multi-frequency ultrasound outperformed single-frequency treatment, likely because multi-frequency ultrasound can generate a more uniform and denser cavitation field, leading to more thorough cell wall disruption. DUSWE achieved the highest yield (4.60%), representing a 33.54% increase compared to HWE. This is attributed to the ultrasonic cavitation effect thoroughly destroying cell structures and releasing phenolic compounds, while subcritical water, with its high temperature and pressure and reduced dielectric constant, exhibits greatly enhanced solubility, efficiently dissolving and rapidly transferring polyphenols, thereby further significantly improving extraction yield. This is consistent with the results reported by Kashtiban et al. [35] for phenolic compounds extracted from grape skins. The combination of ultrasound with subcritical water for extracting bioactive plant compounds is gaining increasing attention.

### 3.3. Active Components

Compared to HWE, most methods showed higher TPC, TFC, and ANT contents (*p* < 0.05), as shown in Figure 3B–D. Subcritical water extraction significantly increased the phenolic content in rapeseed meal, substantially higher than HWE (TPC 39.61 mg/g, TFC 388.19 mg/100 g, ANT 26.03 mg/100 g). Many studies have found that subcritical water extraction yields higher amounts of active components than traditional methods, such as phenolics [14] and cinnamon bark oil. This can be attributed to accelerated mass transfer and increased solubility of polyphenolic compounds under subcritical water conditions due to reduced water polarity. The cavitation, mechanical, and thermal effects of ultrasound can disrupt the cellular structure of rapeseed meal, reduce the particle size of active substances, and improve the mass transfer efficiency of bound components [68,69,70]. Dual-frequency ultrasound treatment outperformed single- and tri-frequency treatments. The shorter bubble collapse time in dual-frequency ultrasound likely produces stronger cavitation effects, thereby enhancing extraction efficiency. Among these methods, DUSWE achieved the highest TPC and TFC, representing increases of 34.99% and 63.12%, respectively, relative to HWE. Its ANT content was 49.56% higher than that of HWE.

### 3.4. Anti-Nutritional Factors

Different extraction methods significantly affected anti-nutritional factors (*p* < 0.05), as shown in Table 2. Compared to traditional hot water extraction, all treatments increased tannin content in the polyphenol extracts. This is because tannins are phenolic compounds, and both ultrasound and subcritical water can promote the dissolution of phenolics, leading to increased content. HWE had the highest phytic acid content (5.36 mg/g), while DUE and DUSWE had the lowest (3.90 and 3.98 mg/g, respectively), representing reductions of 27.24% and 25.75% compared to the HWE group. The HWE group had the highest glucosinolate content (17.09 µmol/g), while DUSWE had the lowest (10.69 µmol/g), a decrease of 37.45%. The structural disruption caused by ultrasound may alter the transfer behavior of phytic acid during subsequent extraction, while high-temperature subcritical water treatment may also affect the stability of glucosinolates. Therefore, the sequential combination of ultrasound and subcritical water extraction may have jointly contributed to the lower measured phytic acid and glucosinolate contents in the purified DUSWE extract. In summary, DUSWE reduced phytic acid and glucosinolate contents in the polyphenol extracts.

### 3.5. Structural Characteristics

**Figure 4:**
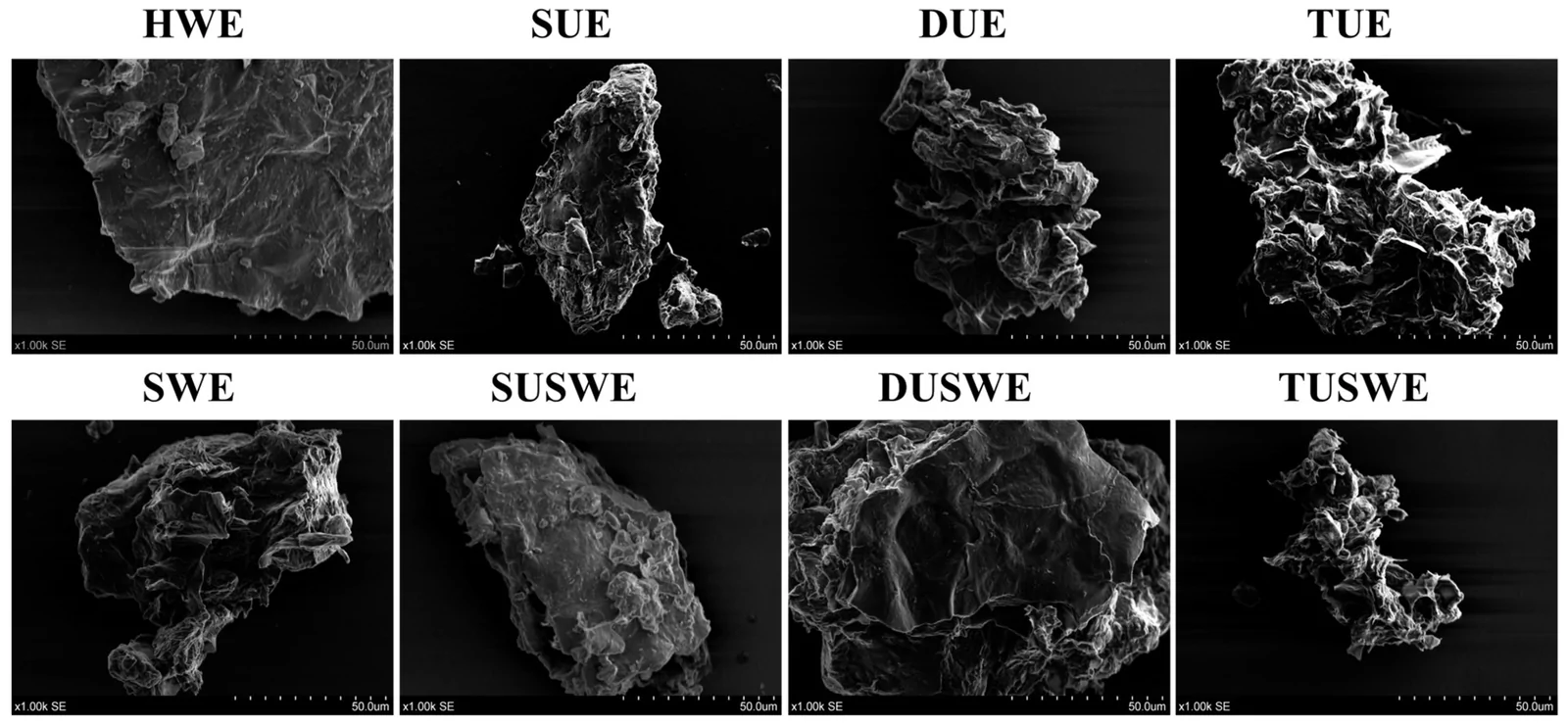
The images of residues after different treatments were observed using a scanning electron microscope.

To investigate the effects of different extraction methods on the microstructure of rapeseed meal residues, scanning electron microscopy was performed, and the results are shown in Figure 4. Compared to the HWE, different treatments altered the structural conformation of the samples [71]. Similar disruption and loosening of the rapeseed meal microstructure after physical field-assisted processing have also been observed in previous studies [72,73]. The HWE residue surface remained relatively intact, dense, and uniformly distributed. Among the ultrasound-treated groups, SUE samples exhibited partially networked chain-like structures with localized wrinkles, fragmentation, and obvious flaking traces at the edges. DUE samples showed further loosened structures with lamellar-like fractures. TUE samples displayed highly fragmented, porous, and loose particles, indicating that the cavitation and mechanical effects of multi-frequency ultrasound intensified with increasing frequency complexity. SWE samples presented internal cracks without complete structural collapse. The combination of ultrasound with subcritical water further exacerbated structural disintegration. Notably, DUSWE-treated rapeseed meal residues were completely fragmented, forming a porous and disordered network structure, which facilitated the release of target components. In contrast, TUSWE samples exhibited large-scale exfoliation, pronounced surface cracking, and exposure of internal layered structures, although excessive disruption might lead to the loss of some target compounds. Unlike DUE, DUSWE combines ultrasound pretreatment with subsequent subcritical water extraction, which may promote solvent penetration and mass transfer. Rapid solvent penetration promotes the release of intracellular compounds, shortens extraction time, and enhances extraction efficiency [74].

### 3.6. In Vitro Antioxidant Capacity

**Figure 5:**
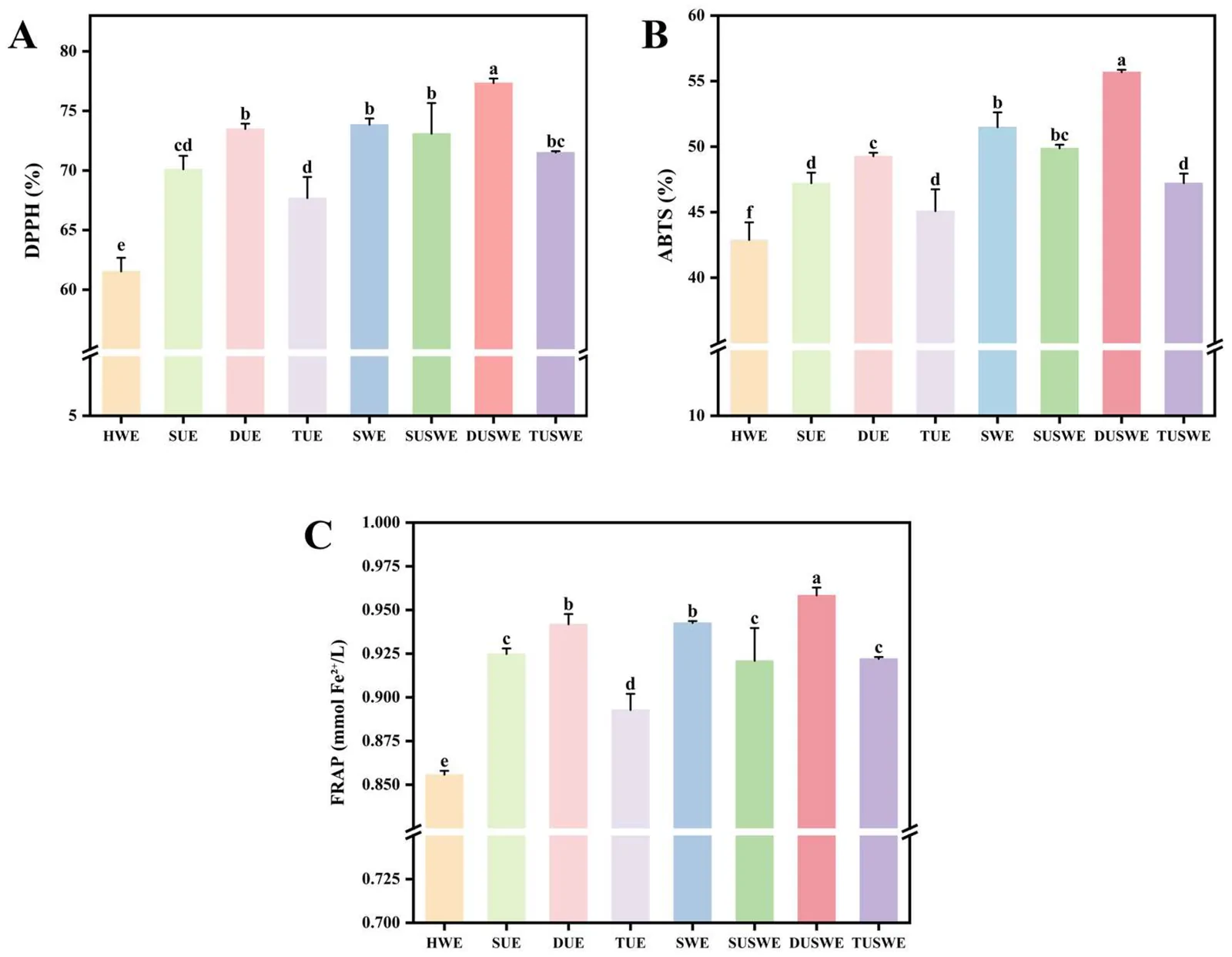
The effect of different methods on the antioxidant properties. (**A**) DPPH; (**B**) ABTS; (**C**) FRAP. Note: HWE: Hot water extraction. SUE: Single-frequency ultrasound-assisted extraction. DUE: Dual-frequency ultrasound-assisted extraction. TUE: Tri-frequency ultrasound-assisted extraction. SWE: Subcritical water extraction. SUSWE, DUSWE, and TUSWE denote single-, dual-, and tri-frequency ultrasound pretreatment followed by subcritical water extraction, respectively. All compositional and in vitro antioxidant results were expressed on a purified freeze-dried extract basis. Data are presented as mean ± SD from three independent extraction batches (*n* = 3). Statistical differences were evaluated using one-way ANOVA followed by the Waller–Duncan multiple-range test. Different lowercase letters within the same panel indicate significant differences among treatments or factor levels (*p* < 0.05).

Different methods showed similar trends in DPPH and ABTS radical scavenging activities (Figure 5A,B). SWE exhibited more significant DPPH and ABTS radical scavenging activities than HWE (*p* < 0.05). Among ultrasound-treated groups, the dual-frequency group (20/40 kHz) showed DPPH and ABTS scavenging rates of 73.29% and 49.29%, respectively, significantly higher than the single-frequency group 40 kHz (70.23%, 47.21%) and the tri-frequency group 20/40/60 kHz (67.69%, 45.02%) [75]. DUSWE exhibited the strongest DPPH and ABTS radical scavenging capacities (77.45%, 55.71%) among all treatments. This trend is consistent with the increases in total phenolic and total flavonoid contents, suggesting that the enhanced in vitro antioxidant capacity of the treated extracts is mainly attributable to the higher content of these phenolic constituents [76,77]. The ferric reducing antioxidant power (FRAP) assay further confirmed this pattern, with DUSWE exhibiting superior ferric reducing ability (Figure 5C), and FRAP values strongly correlated with phenolic constituents [78]. The observed antioxidant differences may reflect both the overall phenolic and flavonoid contents and the structural characteristics of the extracted phenolic constituents. Phenolic hydroxyl groups can contribute to in vitro antioxidant capacity through hydrogen-atom transfer and single-electron transfer, while their number and distribution also influence antioxidant performance [79,80].

## 4. Conclusions

This study is the first to combine multi-frequency ultrasound pretreatment with subcritical water extraction for the preparation of phenolic-enriched extracts from rapeseed meal. Under the conditions investigated, DUSWE resulted in a relatively high dry-extract yield, total phenolic content, and total flavonoid content, together with strong in vitro antioxidant capacity, while the purified extract showed relatively low levels of phytic acid and glucosinolates. Overall, the results indicate that multi-frequency ultrasound pretreatment may improve selected outcomes of subsequent subcritical water extraction and provide a basis for the value-added utilization of phenolic compounds from rapeseed meal. However, this study was conducted using only one rapeseed meal variety and at the laboratory scale. Future studies should further characterize specific phenolic compounds, particularly sinapic acid and its derivatives, using targeted or untargeted metabolomic approaches to clarify how different extraction treatments affect phenolic composition and antioxidant activity.

## Figures and Tables

**Table 1:** Extraction parameters for different methods.

Method	Condition
HWE	Hot water extraction: 100 °C, 12 g/240 mL, 20 min
SUE	Ultrasound: 20 °C, 12 g/240 mL, 20 min, 40 kHz, 168 W
DUE	Ultrasound: 20 °C, 12 g/240 mL, 20 min, 20/40 kHz, 168 W
TUE	Ultrasound: 20 °C, 12 g/240 mL, 20 min, 20/40/60 kHz, 168 W
SWE	Subcritical water: 160 °C, 0.75 g/15 mL (16 times), 15 min
SUSWE	Ultrasound: 20 °C, 0.75 g/15 mL (16 times), 20 min, 40 kHz, 168 W; Subcritical water: 160 °C, 15 min
DUSWE	Ultrasound: 20 °C, 0.75 g/15 mL (16 times), 20 min, 20/40 kHz, 168 W; Subcritical water: 160 °C, 15 min
TUSWE	Ultrasound: 20 °C, 0.75 g/15 mL (16 times), 20 min, 20/40/60 kHz, 168 W; Subcritical water: 160 °C, 15 min

Note: HWE: Hot water extraction, SUE: Single-frequency ultrasound-assisted extraction, DUE: Dual-frequency ultrasound-assisted extraction, TUE: Tri-frequency ultrasound-assisted extraction, SWE: Subcritical water extraction, SUSWE, DUSWE, and TUSWE denote single-, dual-, and tri-frequency ultrasound pretreatment followed by subcritical water extraction, respectively. These treatments were conducted sequentially in two steps. After ultrasound pretreatment, the same slurry was transferred directly to the batch-type subcritical water reactor for subsequent extraction.

**Table 2:** The effect of different methods on the content of anti-nutritional factors.

	Tannins (mg/g)	Phytic Acid (mg/g)	Glucosinolates (μmol/g)
HWE	10.12 ± 0.68 ^e^	5.36 ± 0.08 ^a^	17.09 ± 0.23 ^a^
SUE	12.09 ± 0.13 ^bc^	4.54 ± 0.10 ^cd^	14.28 ± 2.96 ^ab^
DUE	12.50 ± 0.20 ^ab^	3.90 ± 0.14 ^f^	13.26 ± 1.83 ^bc^
TUE	11.38 ± 0.13 ^d^	4.88 ± 0.12 ^b^	14.52 ± 1.36 ^ab^
SWE	12.30 ± 0.10 ^ab^	4.17 ± 0.08 ^e^	13.32 ± 0.65 ^bc^
SUSWE	12.40 ± 0.23 ^ab^	4.41 ± 0.06 ^d^	13.78 ± 0.19 ^b^
DUSWE	12.69 ± 0.17 ^a^	3.98 ± 0.04 ^f^	10.69 ± 0.50 ^c^
TUSWE	11.78 ± 0.04 ^cd^	4.59 ± 0.10 ^c^	14.37 ± 2.07 ^ab^

Note: HWE: Hot water extraction. SUE: Single-frequency ultrasound-assisted extraction. DUE: Dual-frequency ultrasound-assisted extraction. TUE: Tri-frequency ultrasound-assisted extraction. SWE: Subcritical water extraction. SUSWE, DUSWE, and TUSWE denote single-, dual-, and tri-frequency ultrasound pretreatment followed by subcritical water extraction, respectively. All values are expressed per unit mass of purified freeze-dried extract. Data are presented as mean ± SD from three independent extraction batches (*n* = 3), which were purified and analyzed separately. Statistical differences were evaluated using one-way ANOVA followed by the Waller–Duncan multiple-range test. Different lowercase letters within the same column indicate significant differences among treatments (*p* < 0.05).

## Data Availability

The original contributions presented in this study are included in the article. Further inquiries can be directed to the corresponding authors.
